# Phytochemical Analysis and Biological Activity of Three *Stachys* Species (Lamiaceae) from Romania

**DOI:** 10.3390/plants10122710

**Published:** 2021-12-09

**Authors:** Diana Ionela Stegăruș, Ecaterina Lengyel, George Florian Apostolescu, Oana Romina Botoran, Corneliu Tanase

**Affiliations:** 1 National Research and Development Institute for Cryogenics and Isotopic Technologies—ICSI Ramnicu Valcea, 4th Uzinei Street, 240050 Ramnicu Valcea, Romania; diana.stegarus@icsi.ro; 2 Department of Agricultural Sciences and Food Engineering, Lucian Blaga University of Sibiu, Doctor Ion Rațiu 7, 550012 Sibiu, Romania; ecaterina.lengyel@ulbsibiu.ro; 3 Department of Pharmacognosy & Phytotherapy, Faculty of Pharmacy, University of Medicine and Pharmacy of Craiova, 2 Petru Rareş Street, 200349 Craiova, Romania; apostolescugeorge@yahoo.com; 4 Department of Pharmaceutical Botany, “George Emil Palade” University of Medicine, Pharmacy, Sciences and Technology of Târgu Mureș, 38 Gheorghe Marinescu Street, Târgu Mureș, 540139 Mureș, Romania

**Keywords:** antibacterial activity, antioxidant activity, *S. byzantina*, *S. officinalis*, *S. sylvatica*, polyphenols, volatile compounds

## Abstract

Three species of *Stachys* genus (*S. byzantina*, *S. officinalis*, *S. sylvatica*) were investigated in the present study in terms of aromatic profile and total polyphenol content, as well as antibacterial activity and antioxidant capacity. Gas chromatography coupled with flame ionization detection (GC/FID) was used for exploration of the herbal alcoholic extracts. Using statistical analysis, volatile organic compounds (VOCs) and total phenolic chemical fingerprints were compared in order to describe differences and identify putative signature traits of the three *Stachys* species. The results showed that the analyzed *Stachys* extracts have a total polyphenol content being between 197 ± 0.27 mg GAE/g for *S. sylvatica* and 232 ± 43 mg GAE/g for *S. officinalis.* The antioxidant activity was between 444 ± 58 mM Trolox/g (*S. sylvatica*) and 602 ± 75 mM Trolox/g (*S. officinalis*). The volatile compounds identified were mostly sesquiterpenes, followed by monoterpenes and secondary compounds. The most abundant in all three species was germacrene D (21.9% 28–25.2%). The multivariate analysis demonstrated the potential of using plant tissue VOC profiles to discriminate between different *Stachy* species, with a total of 31 VOCs being identified from all three species. Although there were strong similarities among the three species’ VOC profiles, distinctions can be made using chemometric analysis. The microbiological results showed an antimicrobial capacity of all three extracts, especially on Gram-positive bacteria. In addition to increasing consumers’ understanding regarding the health benefits of these *Stachy* species, this investigation contributes to defining and preserving a precious genetic and cultural-historical biodiversity.

## 1. Introduction

In horticulture, quality may be defined as the degree of excellence provided by the combination of various characteristics and traits that provide each plant’s relative value for the intended purpose [1]. Visual appearance, capability to resist different post-harvest processing activities, chemical and nutritional composition, as well as aroma profile, represent principal factors that may be included in this holistic approach [2]. Horticultural breeding has advanced to the point where it is now possible to find fruits and vegetables that have attributes that producers and vendors require, such as high yield, better resistance to pest threats and disease, aesthetic design, and the ability to withstand a wide range of collection and storage operations, among other characteristics. Although several of these agricultural products achieve superior nutritional and flavor attributes, for many products this is very rare [3]. It is still difficult to improve the aroma profile of horticultural plants through breeding techniques, due to the large number of factors that influence the synthesis of volatile and nonvolatile compounds responsible for flavor attributes, including climate and cultural practices [4], agriculture (organic vs. conventional), and pre- and post-harvest processing operations. In general, plants represent a well-known source of natural bioactive molecules in alternative and complementary medicine, and serve as a great resource for many contemporary medications that are produced from natural resources [5]. Various intrinsic and extrinsic variables have been shown to have an impact on plant growth and development; in particular, medicinal plants are strongly affected, as regards the biochemical and physiological functions of their essential oils [6]. Moreover, while the study of plants has been a concern since ancient times in the field of traditional medicine, interest in this field is becoming more intense, due to advanced developments in technology suited to investigating their therapeutic effects. One of the most important properties of plants is their action on the human body [7,8,9,10]. These aspects are highlighted especially through study of the levels and types of bioactive compounds they contain [11,12,13,14] and/or on their biological activity [15,16,17,18].

The biodiversity existing in the temperate and/or Mediterranean area is currently insufficiently studied, in terms of pharmacological potential. In recent years, plant extracts rich in polyphenols have provided a solid basis for the production of alternative pharmaceutical products [19,20]. Plant extracts have been able to offer a lot of opportunities to mankind through their chemical diversity, since they have in their constitution a series of valuable bioactive compounds, including phenolics, flavonoids, iridoids and diterpenoids, that can exhibit significant antifungal, antimicrobial, anti-inflammatory, antioxidant and cytotoxic properties [21,22,23].

The Lamiaceae family is well known for its essential oils, rich in aromatic compounds, with both medical and gastronomic applications [23,24]. *Stachys* species have traditionally been used for therapeutic purposes but also in the cuisine of many peoples in the Mediterranean area as part of their nutritional intake, with tubers rich in protein and carbohydrates [22]. Various *Stachys* species are distinguished by a high concentration of tannins, the extracts being astringent and bitter but with major potential in the treatment of various biliary diseases, diarrhea, gout or varicose veins, and with certain antimicrobial properties [25,26]. 

In Romania, 14 species of *Stachys* have been identified so far, in spontaneous flora, according to the literature [27]. The *Stachys* genus comprises herbaceous plants with simple or branched stems, with opposite leaves, narrow or broad-lanceolate, with corollary flowers grouped (2 to 20) [27]. Active principles identified in the *Stachys* species from Romania include tannins, bitter substances, choline, stachydrins or betaines, which have been recommended for lung diseases and treatments, asthma, diarrhea, gout, varicose ulcers and other conditions [28]. High-performance methods have been used to identify a variety of volatile and non-volatile compounds, flavonoids, monoterpenoids, phenolic acids (caffeic, chlorogenic, p-coumaric), fatty acids and essential oils, choline and betaine [11,25,29], as well as secondary metabolites, among other compounds. Given the fact that the phytochemical composition of these species is responsible for a wide variety of medicinal benefits, the *Stachys* genus has attracted a great deal of interest for the evaluation of its biologically active compounds from various plant parts, including the leaves and fruits. Over 200 chemicals have been identified from this genus, with the majority of them belonging to the key chemical categories of essential oils, polyphenols (e.g., flavonoid derivatives), terpenes and phenolic acids [18,30,31,32,33,34,35].

*Stachys* species are an abundant source of phytochemicals with potential medicinal and commercial uses in a variety of fields. Several *Stachys* cultivars have been produced for use in conventional medicine, the food and pharmaceutical industries and even as ornamental plants as a result of the increasing demand for natural products [18]. The present study was designed taking into account that *Stachys* species are widely used and that there have been a large number of research papers on them; there has been no recent evaluation originating in Romania, however. 

In this context, the research followed two paths: to determine the total polyphenol and volatile chemical content of three species of the *Stachys* genus (*S. byzantina*, *S. officinalis*, and *S. sylvatica*), and to assess their respective antioxidant and antibacterial activity. Using multivariate statistical analysis, the chemical fingerprints of the phenolic compounds (TPCs) and volatile organic compounds were compared in order to define differences and discover possible distinctive characteristics of the three *Stachys* species. Potential compositional characteristics related to antioxidant and antibacterial activity were also explored.

## 2. Materials and Methods

### 2.1. Stachy Samples, Culture Media and Bacterial Strains

The selected species of *Stachys* genus (*S. byzantina*, *S. officinalis*, *S. sylvatica*) were collected randomly, in the summer of 2021 (June and July), during the flowering stage, from the central area of Romania; they were identified and preserved in the Research Center in Biotechnology and Food Engineering Sibiu. The herbs were dried at 35 °C for several days and then shredded. 

The bacterial strains used in this study were *Staphylococcus aureus* (ATCC 25923), *Staphylococcus epidermidis* (ATCC 12228), *Bacillus cereus* (ATCC 9372), *Enterococcus faecalis* (ATCC 19433), *Escherichia coli* (ATCC 8739), *Pseudomonas aeruginosa* (ATCC 10145), *Enterobacter cloacae* (ATCC 43560) and *Proteus mirabilis* (ATCC 29906). The culture media used were Mueller Hinton, Mueller Hinton broth and Cereus selective agar for *Bacillus cereus*.

### 2.2. Chemicals and Reagents

All the chemicals and reagents involved in the extraction processes, total polyphenolic content, antioxidant activity experiments and the chemical standards (1-hexanol, 2-Thujene, 3-octanone, 5-Amino-1-ethylprazole, Allylbenzene, Camphene, Caryophyllene oxide, Decanoic acid, Docosane, Germacrene D, Hexadecane, Hexadecanoic acid, Lavandulol, Limonene, Linalool, Linalool acetate, Nerol, Nerolidol, Ninanal, Tetradecanoic acid, Tricosane, α-Copaene, α-Humulene, α-Ter-pinene, β-Bourbonene, β-Caryophyllene, β-Copaene, β-Cubenene, β-Farnesene, β-Gurjunene, β-Myrcene, β-Pinene) used in the identification of phenolic and aromatic compounds from the extracts were acquired from Sigma Aldrich Company (St. Louis, MO, USA).

### 2.3. Extraction

Fifty grams of plant material was left to soak for 7 days in 500 mL of 96% ethyl alcohol. After maceration, next steps were to filter, centrifuge and concentrate the extract on the water bath at 70 °C, resulting in a dry extract (EU). The dry material was resuspended in 50 mL of distilled water [36]. All subsequent determinations were performed in triplicate in order to obtain the most conclusive results.

### 2.4. Determination of Total Phenolics Content

The total polyphenolic content was measured spectrophotometrically (UV-1900 SHI-MADZU spectrophotometer, Shimadzu Corporation, Kyoto, Japan) using the modified and adapted Folin–Ciocalteu technique [37] using gallic acid as an internal standard. The method involves homogenizing 0.20 mL of sample suspension (extract previously dissolved in distilled water) with 0.80 mL of Folin–Ciocalteu reagent 10% (*v*/*v*) and 1 mL of sodium carbonate (7.5% (*m*/*v*). After a reaction period of 1 h at room temperature (23 °C), the absorbance was read at a wavelength of 750 nm.

The calculation of total polyphenols was done using the formula:

C = c × V/m(1)

where: c—gallic acid concentration determined from the calibration curve in mg/mL; V—volume of extract in mL; m—mass of plant extract in g.

The results are expressed in milligrams of gallic acid equivalent per gram of dry extract (mg GAE/g).

### 2.5. Determination of Volatile Compounds

Analysis of the principal volatile compounds (VOCs) present in the extracts was carried out using gas chromatography (GC) Varian 450-GC, equipped with a flame ionization detector (FID) and a TG-WAXMS capillary column (60 m × 0.32 mm ID × 0.25 µm film thickness). The extracts obtained using the above-mentioned extraction procedures were filtered using a 0.5 m microporous polytetrafluoroethylene filter, and then 1 µL of the filtered extract was directly injected, with a split ratio of 1:10. Three injections were performed for each of the three variants. Helium (He) was used as the carrier gas, with a flow rate of 0.8 mL/min. The column temperature program used for the volatile compound separation increased from 50 °C (held for 1 min) to 110 °C with a rate of 7.5 °C per minute, then up to 160 °C (held for 4 min) with a 14 °C per minute rate, and finally up to 230 °C (held for 8 min). The injector and detector temperatures were set at 260 °C and 290 °C, respectively. The identification of the target compounds was carried out by comparing the retention times of the individual compounds in a chromatogram recorded on a standard solution with the retention times of the same compounds in the chromatograms recorded for the extracted samples, which was done by using the retention times of the individual compounds in the standard solution. It was necessary to dilute the samples and standards before injecting them. It was necessary to carefully dilute the samples and standards before injecting them. Following the completion of this investigation, qualitative and quantitative data were gathered [38]. The percentage of each compound was calculated based on the quantity of each compound determined according to the external standard method.

### 2.6. Determination of Antioxidant Activity

The DPPH (1,1,-diphenyl-2-picrylhydrazyl) method is a spectrophotometric method to quantify antioxidants in complex biological systems commonly used to test the ability of compounds to scavenge free radicals or their ability to donate hydrogen. The method used was as described by Alexandre et al., 2019 [37], amended and adapted. The stock solution was prepared by dissolving in 100 mL of methanol an amount of 24 mg of DPPH, followed by storage in the dark at −20 °C (solution concentration = 600 µM). The working sample was obtained by homogenizing the dry extract in distilled water (1:1); then the working solution was prepared, consisting of a mixture of 90 mL of methanol and 10 mL of stock solution (solution concentration = 60 µM). Twenty-five µL from the sample was allowed to react for 30 min at room temperature (23 °C) in the dark with 175 µL DPPH mixture. The absorbance was read at a wavelength of 515 nm (UV-1900 SHIMADZU spectrophotometer). Distilled water, without extract, was used as a control sample, following the same working procedure. The calibration curve was performed with Trolox, the results being expressed as milligrams of Trolox equivalent per gram of dry extract (mg TE/g EU).

The inhibition percentage (I) was calculated according to the equation:

I (%) = [(Abs A0 − Abs sample): Abs A0] × 100(2)

where Abs A0 represents the control absorbance, and Abs test represent sample and radicals’ reaction absorbance.

### 2.7. Determination of Antimicrobial Activity

The Kirby–Bauer method (diffusimetric) was used to determine the antibacterial activity of the extracts [39]. This approach involves spreading a thin layer of bacterial culture on a solid culture medium (with a concentration of about 0.5 on the MacFarland scale) to determine the antibacterial activity of the extracts. The lyophilized strains were hydrated according to their own protocol, with hydrating liquid. 1 mL of *Staphylococcus aureus* inoculum (ATCC 25923), *Staphylococcus epidermidis* (ATCC 12228), *Enterococcus faecalis* (ATCC 19433), *Escherichia coli* (ATCC 8739), *Pseudomonas aeruginosa* (ATCC 10145), or *Enterobacter cloacae* (ATCC 43560) was deposited and spread on Petri dishes containing Mueller Hinton solid culture medium (Merck KGaA, Darmstadt, Germany), and the inoculum containing *Bacillus cereus* (ATCC 9372) was placed on Cereus selective agar solid culture medium (Merck, Germany). The concentration of the solutions in the plant samples was set at 50 µg/mL. After the culture was wiped, sterile discs with 10 µL, 15 µL, 20 µL of plant extract were applied. After a 24 h incubation at 37 °C, the area of inhibition of the extract was measured with a ruler, including the 6 mm diameter of the disc. The interpretation was made by comparison and/or in mm. Gentamicin and ampicillin in concentrations according to CLSI (Clinical and Laboratory Standard Institute) recommendations were selected as control tests for antibacterial activity on Gram-positive and Gram-negative strains, allowing the establishment of standard methods and procedures in this regard.

### 2.8. Statistical Analysis

The main multivariate statistical analysis approach was based on principal component analysis (PCA) to explain significant associations among quality parameters, content of total phenolic compounds, and VOCs data. Pearson’s correlations (*p* < 0.05 and *p* < 0.01) were used to identify correlations between all of the variables included in the dataset. All the statistical analyses were carried out using the XLSTAT Addinsoft 2014.5.03 software version (Addinsoft Inc., New York, NY, USA).

## 3. Results and Discussions

### 3.1. Total Phenolic Content and Antioxidant Activity

Polyphenols can be extracted by various methods and the efficiency of these processes can be correlated with the plant material and with the structure of the phenolic compounds. Phenolic compounds are present in abundance in the natural environment; specifically, plants tissues produce a high proportion of these molecules. They are thought to have antioxidant capabilities because they include hydroxyl groups in their structure; as a result, it is critical to know their overall quantity in plant samples since this indicates their potential for therapeutic use. The *Stachys* extracts were obtained by applying an ethanol extraction, due to the superior yield compared with other solvents, according to the literature [40,41]. The results obtained can be compared with the standard curve (R2 = 0.9772). *S. officinalis* had a greater total phenolic content than the previously reported extracts of this species [40]. Based on this evaluation, it could be suggested that infusions of *S. officinalis* possess a higher concentration of bioactive compounds than other extracts. Other results achieved here are comparable to those of several different *Stachys* species.

In order to establish the antioxidant activity, the samples were processed according to the DPPH method. The results obtained can be compared with the standard curve (R2 = 0.994). Regarding the ability to inhibit radicals by the DPPH method, it was found that half maximal effective concentration (EC50) values ranged between 11.2 μg/mL for *S. officinalis* and 14.5 μg/mL for *S. sylvatica*. The antioxidant activity was 556 ± 62 mM Trolox/g for *S. byzantina*, 602 ± 75 mM Trolox/g for *S. officinalis* and 444 ± 58 mM trolox/g for *S. sylvatica* (Table 1).

### 3.2. Determination of Volatile Compounds

Chemical contents of three commonly *Stachys* species found in Romania, namely *S. sylvatica*, *S. officinalis*, and *S. byzantina*, were determined and investigated in this study. Table 2 presents the extracted aromatic constituents, each sample presenting a complex composition. Among them, terpenoids comprise the major class of naturally occurring compounds of all the studied extracts. Sesquiterpene hydrocarbons were the most abundant class of constituents found in all taxa, followed by monoterpene hydrocarbons as the second most abundant type. These samples included 29, 30, and 31 volatile compounds, which represented 98.4%, 95.8%, and 97.5% of the total compositions, respectively, according to the results of the GC-MS analysis. *S. sylvatica* and *S. officinalis* extracts had similar chemical profiles. The most abundant groups of compounds were sesquiterpene hydrocarbons (SH) (42.8 and 41.6%), monoterpene hydrocarbons (MH) (36.8 and 31.2 %), and oxygenated monoterpenes (OM) (10.5 and 9.5 %). *S. sylvatica* samples originating from Bulgaria, Italy, Kosovo, Croatia and Hungary were previously investigated by different groups, which reported that sesquiterpene lactones were, as in our case, the major group of substances [42,43]. *S. byzantina* presented the highest concentration of SH and the lowest regarding the content of MH, these proportions having being observed previously in the study conducted by Lashgargahi and Shafaghat [44].

The major constituent of the *Stachy* species was found to be germacrene D [45,46,47]. In the studied *Stachys* species the values of germacrene D were between 25.2% (*S. officinalis*) and 21.9% (*S. sylvatica*). Nevertheless, the presence of other active substances such as limonene, β-pinene, β-cubene, β-caryophyllene and linalool was also identified.

β-caryophyllene is a compound identified in remarkable quantities in all three studied species, the results being between 8.9% (*S. byzantina*) and 13.3% (*S. sylvatica*). It was discovered that the essential oil from a population of *S. officinalis* originating from Serbia contained no β-caryophyllene [16], which was previously found to be a dominant constituent in an essential oil from a community of *S. officinalis* originating from Montenegro [48]. Instead, germacrene D was the predominant compound in the essential oil from a population of *S. officinalis* in Serbia. According to the findings of another study carried out in Serbia [18], sesquiterpene hydrocarbons were the predominant fractions, with germacrene D, β-caryophyllene, and α-humulene being the major components. The primary constituents of *S. officinalis*, according to a research investigation performed on samples originating in Croatia, were germacrene D and €-caryophyllene [32]. It is interesting to observe that germacrene D was detected in significant concentrations in the samples from Italy and Serbia [16,45], while the monoterpene fraction presented low concentrations in all of these investigated samples. In contrast with this finding, *S. sylvatica* originating from Croatia presented a similar concentration of germacrene D and monoterpene hydrocarbons, with α-pinene and β-pinene being the most prevalent components of this fraction as well as in our study. The results of an investigation regarding *S. sylvatica* originating in Kosovo were also consistent with the present findings [49], with the main constituents being represented by α-pinene, β-pinene, and germacrene D. Despite the presence of significant concentrations of germacrene D, αpinene, and β-caryophyllene in *S. sylvatica* collected in Turkey, the ingredient β-pinene was not seen [31]. Regarding monoterpene hydrocarbons, the second most abundant constituents in the essential oil from *S. sylvatica*, comparative results were observed for samples originating from Turkey, with the most abundant compounds being limonene and α-cedrene [24].

β-pinene was quantified at percentages reaching 15.2% (*S. byzantina*). Specific for *S. byzantina* was the high level of β-cubenene of 10.1% identified, compared to the other two species of *Stachys* where this compound does not exceed 0.1%. Another important compound was found in all three species, namely limonene, with levels of 10.8% in *S. byzantina*, 15.7% in *S. officinalis* and 19.5% in *S. sylvatica*. It was observed that this compound is present almost twice as much in *S. sylvatica* compared to *S. byzantina*. In the *S. byzantina* species 1-hexanol compound was not identified, in *S. officinalis* ninanal and tricosane were not identified, while for the *S. sylvatica* 2-thujene, tricosane and β-myrcene were not identified. In a study performed on leaves of *S. byzantina* collected from Western Azarbaijan province, Iran, it was observed that monoterpenes were the predominant fractions, with the most abundant compounds being linalool and 1,8 cineole [44]. These results are different from those obtained in the present study and in the one performed by Lashgargahi and Shafaghat [44], where the principal constituents were represented by the sesquiterpene hydrocarbons, with germacrene D and β-cubenene as most abundant compounds.

Linalool has been identified and quantified at values between 2.5% (*S sylvatica*) and 5.4% (*S. officinalis*), and nerolidol ranges from 2.7% (*S. byzantina*) to 4.7% for (*S sylvatica*), different percentages compared to other studies (0.3–0.8% [32], 0.8% [50]. α- humulene was identified in all three species under study, the values found being between 2.7% (*S. byzantina*) and 1.1% for (*S. sylvatica*), and linalool acetate was between 0.3% and 0.8%. The identified volatile compounds have specificity for each extract, the results obtained being a benchmark for their area of origin.

### 3.3. Multivariate Analysis of the Stachy Species

**Figure 1:**
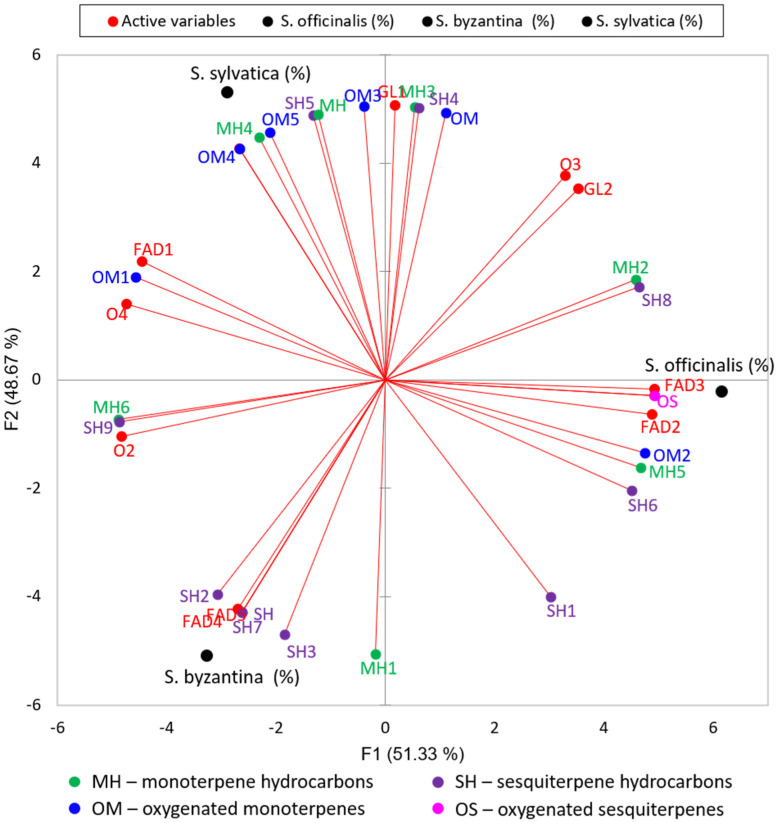
2D-principal component analysis plot of the VOCs in the three *Stachys* species. FAD—fatty acids and fatty acid-derived compounds; GL—green leaf volatiles; MH—monoterpene hydrocarbons; OM—oxygenated monoterpenes; SH—sesquiterpene hydrocarbons; OS—oxygenated sesquiterpenes; O—others.

The VOCs of the extracts of *Stachy* species prepared in the present study were compared using PCA. The applied multivariate statistical analysis helped assess the potential ecological importance of the volatiles data as well as in their interpretation; the findings were statistically supported as a result of the analysis. In order to investigate the potential of VOCs to discriminate among the *Stachys* samples, as well as to identify any important correlations between VOCs, PCA was applied to the GC–MS data. Figure 1 presents a projection of the score and loading values, providing a visual representation of the inter-varietal patterns of similarity or difference, as between the studied Stachys samples, depending on the investigated features. The results of this study indicated that there is a significant variance among native varieties of *Stachys* species.

The two principal components explained 100% of the variation in the dataset, PC1 accounting for 51.33% and PC2 48.67% of the variance. The three *Stachys* samples were clearly dispersed along the PCs in the PCA plot. *S. byzantina* and *S. sylvatica*, in particular, exhibit negative PC1 scores (but negative and positive PC2 scores, indicating that these samples are somehow distinct), and were positioned on the left side of the figure, whilst *S. officinalis* was plotted on the other side. The absolute loading scores (LV ≥ 0.8) were assigned as cut-off in order to explain the maximum variance along the PCs [51], allowing 14 loadings to be selected as the most differentiating components on PC1 (FAD1, FAD2, FAD3, O2, O4, MH2, MH5, MH6, OM1, OM2, OS1, SH6, SH8, and SH9) and 13 loadings with high correlation along PC2 (FAD4, FAD5, GL1, MH1, MH3 (Table 2, Figure 1). Within the most differentiating components on PC1, MH6, OM1 and SH9 terpenoids were detected in *S. byzantina* and *S. sylvatica* in high concentrations; while the *S. officinalis* sample was characterized by relative high amounts of MH1, SH3 and SH7. The presence of other VOCs was found to be similar in all the *Stachys* samples (Table 2). If the variables that separated the samples around F1 are taken into account, the PCA results may support the hypothesis of Lazarevic et al., suggesting that the proportion of total composition of certain molecules, such as oxygenated terpenes like caryophyllene oxide, or oxygenated compounds of terpenoid origin, is influenced by eco-geographic factors [18]. Based on their conclusions, ecological factors on the Balkan Peninsula may influence the amount of terpenoid oxygenation in samples of *S. officinalis* plants, for example, by expressing the genes of the enzymes involved.

### 3.4. Antibacterial Activity

**Figure 2:**
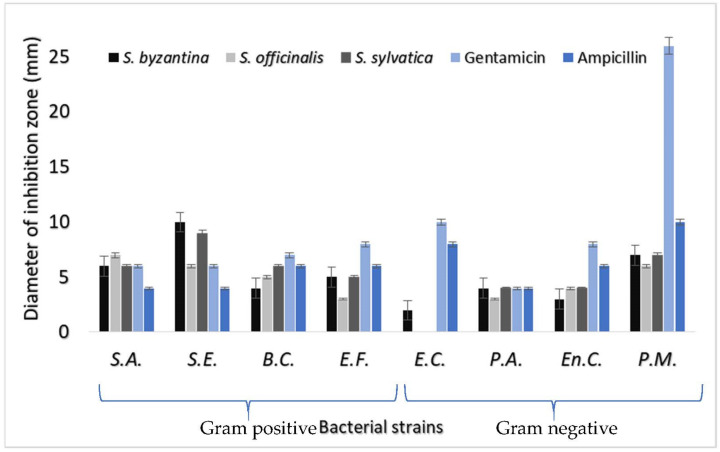
Antibacterial activity of *Stachys byzantina*, *S. officinalis* and *S. sylvatica* extracts on S.A.: *Staphylococcus aureus* (ATCC 25923), S.E.: *Staphylococcus epidermidis* (ATCC 12228), B.C.: *Bacillus cereus* (ATCC 9372), E.F.: *Enterococcus faecalis* (ATCC 19433), E.C.: *Escherichia coli* (ATCC 8739), P.A.: *Pseudomonas aeruginosa* (ATCC 10145), En.C.: *Enterobacter cloacae* (ATCC 43560), P.M.: *Proteus mirabilis* (ATCC 29906).

Plants with antibacterial potential are currently attracting the attention of scientists, due to the fact that many studies reveal the easy assimilation of valuable natural compounds by the body compared to drugs obtained synthetically [52]. The antibacterial activity of the three selected *Stachys* species is particularly noticeable. As can be seen in Figure 2, the measured zones of inhibition were between 3 and 10 mm for Gram-positive bacteria and up to 7 mm for Gram-negative bacteria. The *Staphylococcus aureus* strain (ATCC 25923) showed more pronounced inhibition by *S. officinalis* extract, where the mean values were 7 mm, while the *Staphylococcus epidermidis* strain (ATCC 12228) showed a much more significant area of inhibition of up to 10 mm in the case of extracts of *S. byzantine*. These values are higher than those recorded for the controls (ampicillin or gentamicin). In addition, from the spectrum of Gram-positive bacteria it can be stated that the strains of *Bacillus cereus* (ATCC 9372) and *Enterococcus faecalis* (ATCC 19433) experienced antibacterial effects with a zone of inhibition measured in a range of 3–6 mm for all three extracts taken in the study. These values are lower than those recorded for the controls (ampicillin or gentamicin).

Regarding the antibacterial action of the three *Stachys* extracts (*S. byzantina*, *S. officinalis*, *S. sylvatica*) on Gram-negative bacteria, it was found that they present different inhibition values. The values recorded for the three extracts were lower compared to those recorded for the controls (ampicillin and gentamicin). The exception was the values observed for *P. aeruginosa*, where these were approximately equal to those of the controls (ampicillin and gentamicin). Thus, the antibacterial effect was minor on the *Escherichia coli* strain (ATCC 8739), with values between 2 mm in the case of *S. byzantina* extract. In the case of the *Proteus mirabilis* strain (ATCC 29906), the most significant values of the antibacterial effect were noticed in the case of *S. byzantina* and *S. sylvatica* extracts where the measured inhibition area exceeded 7 mm. Both *S. officinalis* and *S. sylvatica* extract had an inhibitory effect on the *Enterobacter cloacae* strain (ATCC 43560) of over 4 mm, while *S. byzantina* extract had an effect of around 3 mm. The reaction of *Stachy* extracts regarding bacterial cultures varies, being mostly influenced by the chemical profile, molecular structure of the major components, structural properties and quantities [53]. Also, the antibacterial activity is determined by the complex formation and interactions between major and minor VOC components. Considering previous screenings focused on antimicrobial activity of medicinal plants, our results are in agreement presenting a stronger antibacterial activity against Gram-positive strains and moderate against Gram-negative strains [54]. 

In the current study, the presence of phenols and oxygenated terpenes may explain the inhibition of bacteria, as terpenes such as linalool, neronidol and caryophyllene oxide are more active than terpenoids such as β-pinene and β-caryophyllene hydrocarbons. Due to their limited water solubility, hydrocarbon derivatives have reduced antibacterial activity, limiting their dispersion through the substrate. This inactivity is strongly related to their reduced hydrogen binding capacity and water solubility, whereas oxygenated molecules have a stronger bonding capability [54]. Generalized resistance of Gram-negative bacteria such as *Proteus mirabilis* is attributed to the existence of a hydrophilic outer membrane containing a hydrophilic polysaccharide chain that functions as a hydrophobic barrier against extracellular essential oils [55]. The influence of natural phenolic compounds produced by plants on Gram-negative bacteria biofilm growth has recently been demonstrated [56], but no systematic study regarding the correlation between microbial limitation and total phenolic content of aromatic plants and herbs has been conducted.

## 4. Conclusions

The bioactive compounds from three *Stachys* extracts presented significant values with regard to antioxidant capacity. The antioxidant activity leads to the possibility of their use in the pharmaceutical and agri-food industry. Through the GC-MS analyses performed, over 39 volatile compounds were identified, specific to all species. The most important group of volatile compounds is part of the group of oxygenated and hydrogenated sesquiterpenes, followed by monoterpenes and other non-terpenoid compounds. This study also demonstrated the potential of using plant tissue VOC profiles to discriminate between different *Stachys* species, with a total of 31 VOCs being identified from all three species. Although there were strong similarities among the three species’ VOC profiles, distinctions can be attempted using chemometric analysis. Results from this study provide a feasible and useful method to rapidly classify *Stachys* plants, indicating a great similarity regarding the profile of volatile compounds among *S. officinalis* from South East Europe, even if essential oil constituents differ noticeably according to the individual genetic mutability, different portions of the herb and growth periods. The content and distribution of these components varied among the studied species, owing to several extrinsic factors affecting secondary metabolite composition. Therefore, in order to obtain a high level of scientific and experimental findings and verify the genuine efficacy and safety of plant products that have been used by humans for millennia, additional well-designed investigations are needed [51]. The antimicrobial tests performed showed that the extracts of *S. byzantine*, *S. officinalis* and *S. sylvatica* showed antibacterial activity especially against Gram-positive microorganisms, with the effect being lower in Gram-negative ones. The most relevant results were obtained in the species *S. byzantina* and *S. sylvatica* against *S. epidermidis* and *P. mirabilis*. The traditional use of *Stachys* species derives from known medicinal effects, so the identification and quantification of bioactive compounds lead to multiple possibilities of exploiting these qualities for health and as natural remedies. In addition to increasing consumers’ understanding regarding the health benefits of these *Stachys* species, this investigation provides a means to protect a valuable genetic and cultural heritage.

## Figures and Tables

**Table 1:** Total amount of phenolic compounds and radical scavenging activity in *S. byzantina*, *S. officinalis* and *S. sylvatica*.

Plant Sample Extract	TPC (mg GAE/g) ± SD *	DPPH Scavenging Activity (%) ± SD *	DPPH (EC50) μg/mL ± SD *	TE (mM/g Dry Extract) ± SD *
*Stachys byzantina*	222 ± 0.34	63.8 ± 0.3	12.7 ± 0.01	556 ± 62
*Stachys officinalis*	232 ± 0.43	65.2 ± 0.5	11.2 ± 0.01	602 ± 75
*Stachts sylvatica*	197 ± 0.27	53.1 ± 0.2	14.5 ±0.01	444 ± 58

* TPC—total polyphenol content; GAE—gallic acid equivalent; DPPH—2,2-diphenyl-1-picrylhydrazyl; TE—Trolox equivalent; SD—standard deviation.

**Table 2:** The profile of volatile compounds from the three *Stachys* species: *S. byzantina*, *S. officinalis*, *S. sylvatica*.

Compound Name	*S. byzantine* (%)	*S. officinalis* (%)	*S. sylvatica* (%)	Compound Classification
Docosane	0.7	0.4	0.9	FAD_1_
Hexadecanoic acid	0.2	1.1	0.1	FAD_2_
Tetradecanoic acid	0.1	0.3	0.1	FAD_3_
Tricosane	0.2	0	0	FAD_4_
Hexadecan-1-ol	0.2	0.1	0.1	FAD_5_
1-hexanol	0	0.1	0.2	GL_1_
Decanoic acid	0.2	1	0.8	GL_2_
3-octanone	0.2	0.1	1.2	O_1_
5-Amino-1-ethylprazole	0.5	0.1	0.4	O_2_
Allylbenzene	0.4	1.5	1.3	O_3_
Ninanal	0.3	0	0.4	O_4_
2-Thujene	0.2	0.1	0	MH_1_
Camphene	0.6	1.1	0.8	MH_2_
Limonene	10.8	15.7	19.5	MH_3_
α-Terpinene	2.1	2.1	2.2	MH_4_
Β-Myrcene	0.5	1.7	0	MH_5_
Β-Pinene	15.2	10.5	14.3	MH_6_
Lavandulol	0.5	0.1	0.7	OM_1_
Linalool	3.2	5.4	2.5	OM_2_
Linalool acetate	0.3	0.5	0.8	OM_3_
Nerol	0.8	0.7	1.8	OM_4_
Nerolidol	2.7	2.8	4.7	OM_5_
Caryophyllene oxide	1.9	5.7	1.8	OS_1_
Germacrene D	24.6	25.2	21.9	SH_1_
α-Copaene	0.9	0.1	0.2	SH_2_
α-Humulene	2.7	1.4	1.1	SH_3_
β-Bourbonene	0.1	1.2	2	SH_4_
β-Caryophyllene	8.9	9.9	13.3	SH_5_
β-Copaene	1.9	2.4	1.6	SH_6_
β-Cubenene	10.1	0.1	0.1	SH_7_
β-Farnesene	0.2	1	0.5	SH_8_
β-Gurjunene	1.3	0.3	1.1	SH_9_
TOTAL (%)	97.5	95.8	98.4	
MH	29.4	31.2	36.8	
OM	7.5	9.5	10.5	
SH	50.7	41.6	41.8	
OS	1.9	5.7	1.8	

FAD—fatty acids and fatty acid-derived compounds; GL—green leaf volatiles; MH—monoterpene hydrocarbons; OM—oxygenated monoterpenes; SH—sesquiterpene hydrocarbons; OS—oxygenated sesquiterpenes; O—others.

## Data Availability

Data is contained within the article.
